# Search for missing symmetry in the Inorganic Crystal Structure Database (ICSD)

**DOI:** 10.1107/S2052520624008229

**Published:** 2024-09-17

**Authors:** Maxim Avdeev

**Affiliations:** a Australian Centre for Neutron Scattering Australian Nuclear Science and Technology Organisation New Illawarra Road Lucas Heights NSW2234 Australia; b School of Chemistry University of Sydney Sydney NSW2006 Australia; Moscow State University, Russian Federation

**Keywords:** Inorganic Crystal Structure Database (ICSD), symmetry, space groups, centrosymmetric structures

## Abstract

An exhaustive search for missing symmetry was performed for 223 076 entries in the ICSD (2023-2 release). Approximately 0.65% of them can be described with higher symmetry than reported. Out of the identified noncentrosymmetric entries, ∼74% can be described by centrosymmetric space groups; this has implications for compatible physical properties. It is proposed that the information on the correct space group is included in the ICSD.

## Abstract 2

An exhaustive search for missing symmetry was performed for 223 076 entries of the ICSD. About 0.65% of the entries can be described with higher symmetry than reported. It is proposed that the information is included in the ICSD.

## 1. Introduction

Describing crystal structures with unnecessarily low symmetry is a well known outcome of many crystal structural studies. For hundreds of compounds, the space groups have been corrected, most notably by Richard Marsh and coworkers (Marsh & Schomaker, 1979 ▸; Marsh, 1980 ▸; Marsh & Schomaker, 1981 ▸; Herbstein & Marsh, 1982 ▸; Marsh & Herbstein, 1983 ▸; Marsh, 1983 ▸, 1984 ▸; Marsh & Slagle, 1985 ▸; Marsh, 1986*a* ▸,*b* ▸,*c* ▸; Marsh *et al.*, 1986 ▸; Marsh & Schaefer, 1986 ▸; Marsh, 1987 ▸; Marsh & Schomaker, 1987 ▸; Marsh, 1988*a* ▸,*b* ▸,*c* ▸,*d* ▸,*e* ▸; Marsh & Herbstein, 1988 ▸; Marsh & Robinson, 1988 ▸; Marsh & Slagle, 1988 ▸; Kapon *et al.*, 1989 ▸; Marsh, 1989*a* ▸,*b* ▸,*c* ▸,*d* ▸,*e* ▸,*f* ▸,*g* ▸,*h* ▸,*i* ▸, 1990*a* ▸,*b* ▸*c* ▸,*d* ▸,*e* ▸; Marsh & Meyer, 1990 ▸; Marsh, 1991*a* ▸,*b* ▸,*c* ▸, 1992 ▸, 1993*a* ▸,*b* ▸,*c* ▸,*d* ▸, 1994 ▸, 1995 ▸; Marsh & Bernal, 1995 ▸; McCarroll *et al.*, 1995 ▸; Connick *et al.*, 1996 ▸; Marsh, 1996 ▸, 1997 ▸; Herbstein & Marsh, 1998 ▸; Marsh, 1998 ▸; Leclaire *et al.*, 2001 ▸; Marsh & Spek, 2001 ▸; Marsh, 2002 ▸; Marsh *et al.*, 2002 ▸; Marsh, 2004 ▸, 2005 ▸; Marsh & Clemente, 2007 ▸; Henling & Marsh, 2014 ▸) and others (Jones, 1984 ▸; Baur & Tillmanns, 1986 ▸; Baur & Kassner, 1992 ▸; Clemente & Marzotto, 2003 ▸; Clemente, 2003 ▸; Clemente & Marzotto, 2004 ▸; Clemente, 2005 ▸).

Until the late 1980s, the corrections were done by hand after examining the published structures or performing structure redeterminations using the original diffraction data. The development of dedicated software (Le Page, 1987 ▸, 1988 ▸; Spek, 2020 ▸; Stokes & Hatch, 2005 ▸; Capillas *et al.*, 2011 ▸) significantly simplified the process and nowadays testing for missing symmetry is a standard step in the determination of new crystal structures. However, the efforts of correcting space groups for the previously published structures were mostly focused on organic materials. The Inorganic Crystal Structure Database (Zagorac *et al.*, 2019 ▸), which is one of the main sources of experimental crystal structural information in the field of inorganic solid-state chemistry, was never exhaustively analysed even though the survey of structures published in *Acta Crystallographica* and *Crystal Structure Communications* led to an estimate that about 3% of all the published structures may have been described with too low symmetry (Baur & Tillmanns, 1986 ▸). The largest reported effort is the analysis of 54 000 ICSD entries, the subset of the *AFLOW* repository (http://www.aflow.org/) (Hicks *et al.*, 2018 ▸); however, the focus of the report was on testing the capabilities of the *AFLOW-SYM* package.

Therefore, an exhaustive search for missing symmetry for all the entries in the Inorganic Crystal Structure Database was undertaken in this work. The motivation was not only to simply set the crystallographic record straight. The question of the correct space group, *e.g.* centrosymmetric versus noncentrosymmetric, is critical for the compatibility of physical properties, *e.g.* piezo-, ferroelectric, nonlinear optical effects *etc*. The recent rapid adoption of unsupervised machine learning techniques to process large data sets critically relies on the quality of the used data. For instance, a crystal structure, for which a centre of symmetry was overlooked, may be incorrectly identified as a candidate to possess piezoelectric or other physical properties, allowed only in noncentrosymmetric space groups.

## 2. Analysis details

There are several software codes capable of finding a space group from unit-cell parameters and atomic coordinates, *e.g.PLATON* (Spek, 2020 ▸), *FINDSYM* (Stokes & Hatch, 2005 ▸), *spglib* (Togo & Tanaka, 2018 ▸), *AFLOW-SYM* (Hicks *et al.*, 2018 ▸), *Findsym* (*Materials Studio*; Dassault Systèmes, 2022 ▸), but not all of them can automatically import and process large numbers of Crystallographic Information Files (CIFs). They also define and use tolerances differently on atomic coordinates and unit-cell parameters to identify a space group, as reviewed by Hicks *et al.* (2018 ▸). Therefore, three codes were selected for cross-validation of the results, *i.e.* the built-in function of *MaterialsScript* in *Materials Studio* (Dassault Systèmes, 2022 ▸), which we previously used for high-throughput analysis (Sale & Avdeev, 2012 ▸; Avdeev *et al.*, 2012 ▸), *FINDSYM* (version 7.1.4) (Stokes & Hatch, 2005 ▸), and *AFLOW-SYM* (version 3.2.13) (Hicks *et al.*, 2018 ▸).

All 223 076 entries of the ICSD (release 2023.2) were processed at a tolerance of 10^−6^ Å on the distance between the reported positions of atoms and those in the corresponding symmetrized structure, which was deemed to be sufficiently tight, given that the ICSD entries report the experimentally determined values with substantially lower precision, as illustrated in Fig. S1. Needless to say, the higher symmetry structure, if detected, corresponds to the very same temperature and pressure reported for the original structure, since changes in external physical conditions typically cause variation of atomic positions and unit-cell parameters far beyond the 10^−6^ Å range.

Unfortunately, the *AFLOW-SYM* code was unable to process more than 50 000 CIFs with mixed occupancies, *i.e.* with zero interatomic distance between atoms residing on the same site, which generated the error ‘The tolerance cannot be larger than the minimum interatomic distance’. The other two codes, *Materials Studio* and *FINDSYM*, also failed to process some of the CIFs, but for much smaller numbers, 861 and 5918, respectively, mostly due to failures to parse the CIF content. Nevertheless, out of 223 076 CIFs only 72 could not be automatically processed at least by one of the codes, mostly due to typos. These 72 CIFs were manually processed one-by-one. The files with typos were corrected and analysed and only 38 could not be processed at all due to missing values of the atomic coordinates. The remaining CIFs have been automatically analysed by all three, two, or at least one, of the codes (148 222, 70 892, and 3 201, respectively).

## 3. Results and discussion

As a result of the analysis, 1 458 entries (1 214 unique compositions) were identified, which can be described with symmetry higher than reported, *i.e.* ∼0.65% of the total, which is substantially lower than ∼3% estimated previously (Baur & Tillmanns, 1986 ▸); however, see the statistics versus time analysis presented below. The complete list is provided in a spreadsheet in the supporting information.

**Figure 1:**
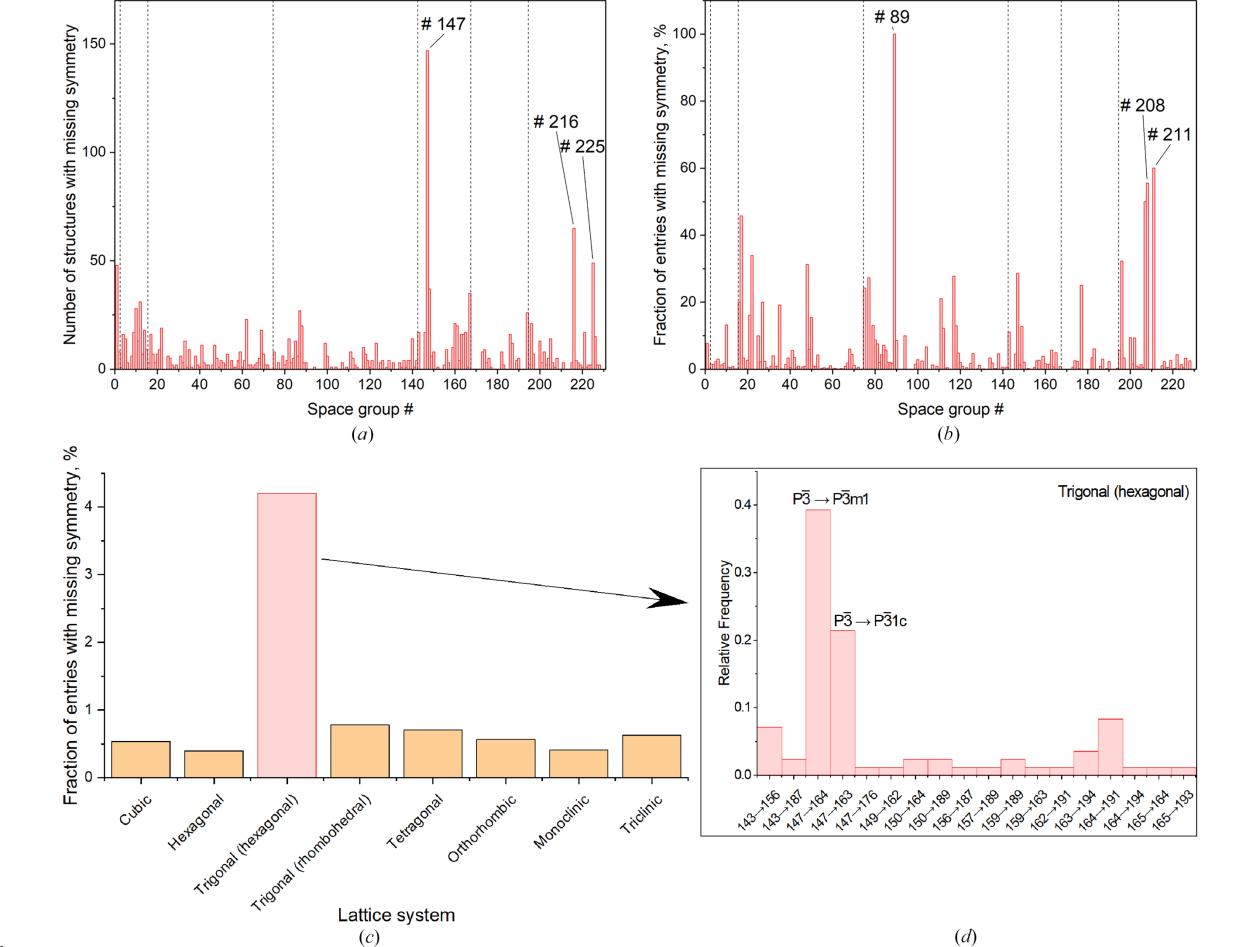
(*a*) The count of space groups with missing symmetry; (*b*) normalized by the number of the entries with the corresponding space group type in the ICSD, *i.e.* divided by the numbers shown in Fig. S2. Labels show the top three. Dashed lines delineate from left to right: triclinic, monoclinic, orthorhombic, tetragonal, trigonal, hexagonal and cubic space groups. (*c*) Fraction of ICSD entries with missing symmetry by lattice system. (*d*) Breakdown of the statistics for the trigonal hexagonal lattice system by space group types in the format ‘original space group in the ICSD → higher-symmetry space group consistent with the structure’.

Next, we explore whether there are any patterns in the distribution of those structures by symmetry and over time. In absolute numbers, the space group types No. 147 (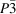), No. 216 (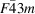) and No. 225 (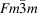) appear to be the most common groups with missing symmetry [Fig. 1 ▸(*a*)]. However, once normalized by the corresponding number of the entries for each group type in the ICSD (illustrated in Fig. S2), it becomes clear that the higher symmetry was often missed for the structures with rare space group types [Fig. 1 ▸(*b*)]. The top three space group types on the normalized scale are No. 89 (*P*422), No. 211 (*I*432) and No. 208 (*P*4_3_22), with only three, five and nine entries in the ICSD, respectively. The case of the space group type No. 89 (*P*422) particularly stands out, as all the three ICSD entries are fully consistent with the space group No. 123 (*P*4/*mmm*), and is a good example of when structures which are effectively centrosymmetric are reported as noncentrosymmetric. Out of the 1458 identified entries, 651 are noncentrosymmetric, 481 of which, *i.e.* ∼74%, can be described by centrosymmetric space groups. Further grouping the entries with missing symmetry by lattice system suggests that the trigonal crystal system is the most affected [Fig. 1 ▸(*c*)]. It should be emphasized that the analysis presented above deals only with self-consistency of the symmetry description for a given structure, not with the question of whether the original study correctly analysed the diffraction data and adequately dealt with all the pitfalls of structure determination, *e.g.* twinning, neglected superstructure reflections, *etc* (Müller, 2013 ▸). Also, it should be clear that the selected very tight tolerance leads to extremely conservative analysis, which identifies only the structures with the atoms on special Wyckoff sites or at a distance within a very small fraction of the reported standard uncertainty (s.u.) from the position in the corresponding higher-symmetry structure. Relaxing the tolerance to the level comparable with the reported s.u.’s (Fig. S1) would yield many more structures consistent with higher symmetry. For example, the study specifically searching for overlooked trigonal symmetry in monoclinic structures (Cenzual *et al.*, 1990 ▸) identified eight cases, including PbTe and CaGa_6_Te_10_, originally reported in space groups *C*2/*m* and *C*2, respectively. Indeed, these two structures can be described in space groups 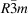 and *R*32 within the tolerances of ∼0.003 Å and ∼0.035 Å, respectively. However, to decide whether the deviation from higher symmetry is statistically significant would require reanalysing the original dataset or new experimental study. Therefore, in this work only the structures consistent with higher symmetry at the level well below the reported precision (Fig. S1) are presented. This approach is probably one of the reasons why the trends illustrated in Fig. 1 ▸ differ from the previous compilation of symmetry correction in 221 structures (Baur & Kassner, 1992 ▸), which found space group *Cc* (No. 9) to be the most represented. Another possible explanation is that low-symmetry structures simply received more attention and were more frequently revisited, which biased the Baur & Kassner (1992 ▸) survey results. In this study we find that the highest fraction of the ICSD entries with overlooked symmetry belongs to the trigonal lattice system [Fig. 1 ▸(*c*)] and, in particular, to the space group type 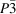 (No. 147), in which mirror and glide planes are apparently often overlooked, and the structures should be instead described in 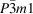 (No. 164) and 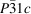 (No. 163) [Fig. 1 ▸(*d*)].

It should also be noted that some of the identified structures were already revisited, *e.g.* in the work by Cenzual *et al.* (1991 ▸) space groups for about 30 structures were revised, using the early *MISSYM* software (Le Page, 1988 ▸). Our analysis produced identical corrections of the space groups, which cross-validates both studies. The structures reported in Cenzual *et al.* (1991 ▸) are indicated by a comment in the spreadsheet in supporting information, except for MgAu_3–*x*_ (ICSD No. 58545), V_6_C_5_ (ICSD No. 654841), and Zr_4_Al_3_ (ICSD No. 150529), for which the tolerance required to increase symmetry is substantially higher than the 10^−6^ Å threshold adopted in this work, *i.e.* ∼0.19, 0.007 and 0.0003 Å, respectively.

**Figure 2:**
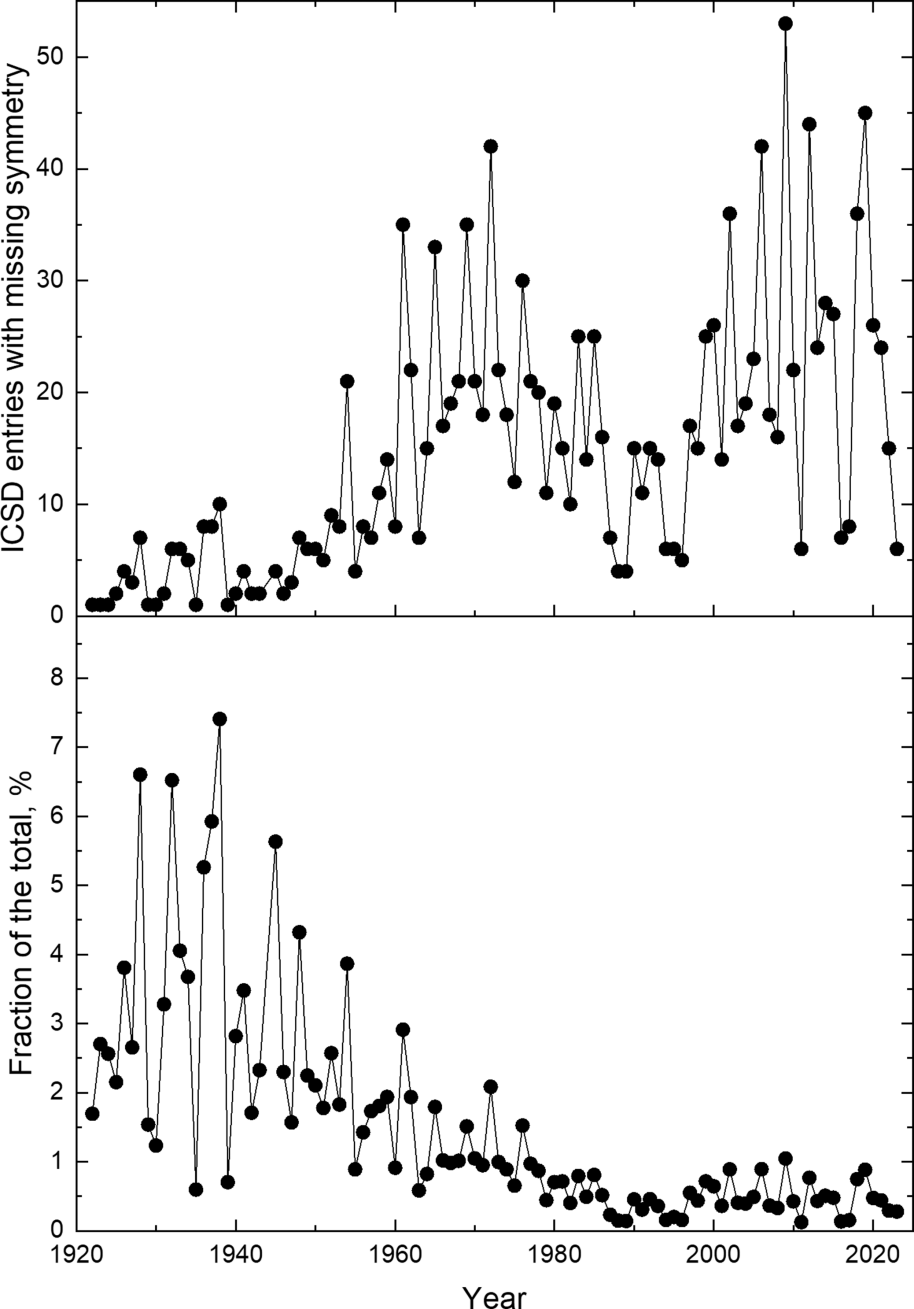
(Top) Number of ICSD entries with missing symmetry versus time; (bottom) normalized to the total number of entries versus time, *i.e.* divided by the numbers shown in Fig. S3.

Finally, the evolution of reporting structures with missing symmetry with time is illustrated in Fig. 2 ▸. Although the number of such entries increased with time, the total number of entries increased at a faster pace (Fig. S3) and around 1980s the fraction of the structures reported with missing symmetry stabilized at ∼0.5%. Development of the algorithms for symmetry search around that time is probably the main factor. At the same time, the fact that the structures with overlooked symmetry still get reported likely reflects the over reliance on modern diffractometers with computer software that determines the space groups using automated routines with default settings. When used blindly, the programs may misinterpret twinning, reject weak reflections such as superstructure reflections *etc.*, all with the consequence of incorrect space-group assignment (Müller *et al.*, 2021 ▸). Missing symmetry identified in the resulting model may be a good indicator to revisit not only the space group but all the steps of the data analysis. The bottom line is that despite all the progress in hardware and software, human competency remains a vital component and the investigator should be able to critically assess computer program output and take advantage of recommendations on how to avoid the pitfalls, which are widely available in crystallography textbooks and numerous journal publications, *e.g.* Baur & Tillmanns (1986 ▸), Baur & Kassner (1992 ▸), Marsh (1995 ▸).

## 4. Conclusions

At present, testing for missed symmetry is largely done automatically by structure determination software. For much of the historical structural information for organic materials the search was carried out by Marsh and co-workers. In contrast, for inorganic structures such analysis was never exhaustively performed, although the number of crystal structures described with unnecessarily low symmetry was estimated at ∼3% (Baur & Tillmanns, 1986 ▸). In this work, search for missing symmetry in 223 076 entries in the Inorganic Crystal Structure Database (release 2023-2) was performed and 1 458 entries (∼0.65%) were identified which can be described by higher symmetry than reported. Correcting symmetry is important for unsupervised high-throughput analysis of the ICSD with machine learning. For instance, ∼74% of the 651 identified noncentrosymmetric structures are consistent with centrosymmetric space groups, which determines what physical properties can be expected, *e.g.* piezoelectricity, *etc*. Therefore, it is proposed that a note for each crystal structure that is compatible with higher symmetry to be added in the ICSD.

## Supplementary Material

Figures S1-S3, and Table S1. DOI: 10.1107/S2052520624008229/yh5036sup1.pdf

Spreadsheet. DOI: 10.1107/S2052520624008229/yh5036sup2.xlsx
