# Supplementary material for: Search for missing symmetry in the Inorganic Crystal Structure Database (ICSD)
Source: Acta Crystallogr B Struct Sci Cryst Eng Mater. 2024 Sep 17;80(Pt 5):451–5. doi: 10.1107/S2052520624008229 (PMC11457106; doi:10.1107/S2052520624008229)
Supplement: Supplementary file 1 [file b-80-00451-sup1.pdf]

STRUCTURAL SCIENCE
CRYSTAL ENGINEERING
MATERIALS

Volume 80 (2024)

Supporting information for article:

Search for missing symmetry in the Inorganic Crystal Structure Database (ICSD)

Maxim Avdeev

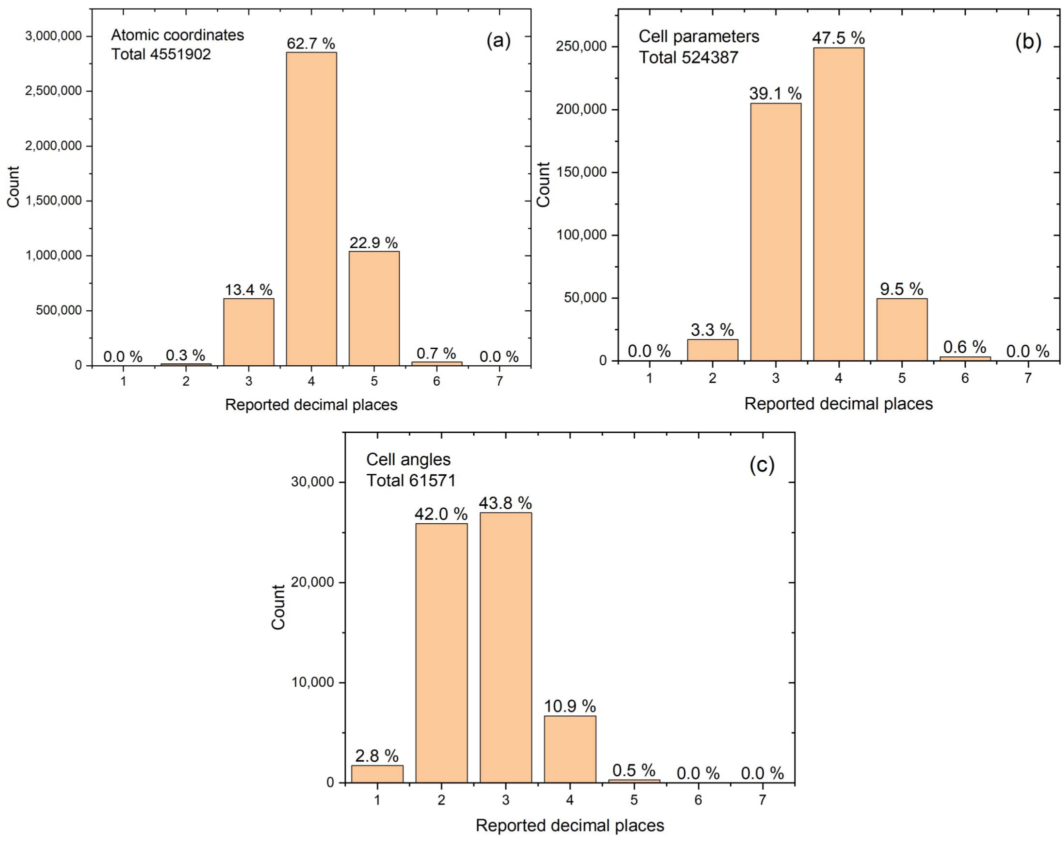

Figure S1 Distribution of the reported decimal places, where estimated standard deviation is given for the last digit, for atomic coordinates (a), unit cell parameters (b), and unit cell angles (c)

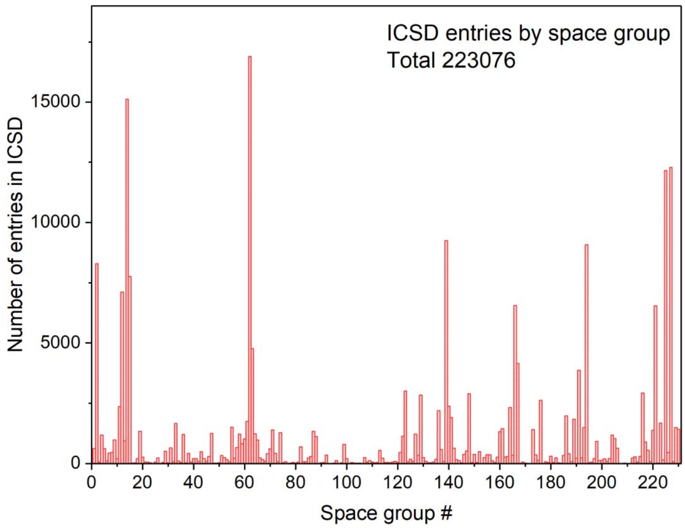

Figure S2 Distribution of the ICSD entries by space group.

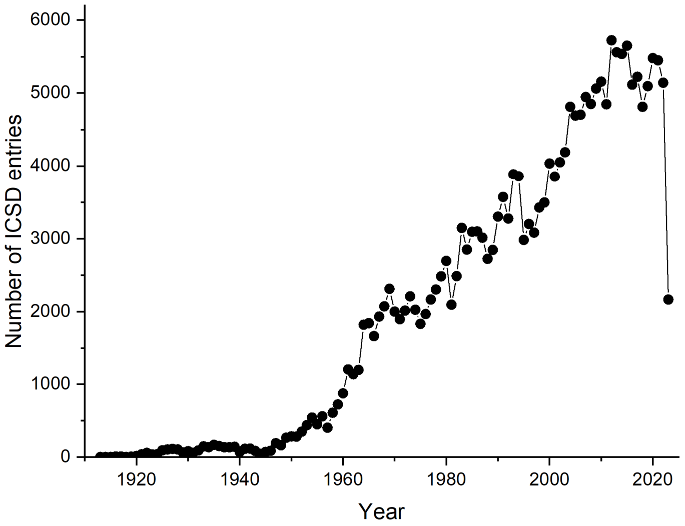

Figure S3 Number of entries added to the ICSD each year vs. time.

Table S1 Commands used to run MaterialsScript, FINDSYM, and AFLOW-SYM.

Software	Command
MaterialsScript	Analysis was run on a Windows 10 desktop using the MaterialsScript with the following commands<br>Tools->Symmetry->ChangeSettings([PositionTolerance => 1e-6]);<br>Tools->Symmetry->FindSymmetry->DistinguishProperty("Occupancy")="Yes";
FINDSYM	Analysis was run from command line under Linux using binaries downloadable from https://stokes.byu.edu/iso/isolinux.php<br>1. cifs were converted into input (.inp) files with findsym_cifinput distributed with findsym<br>2. latticeTolerance and atomicPositionTolerance were set in the input file to 1e-6<br>3. findsym was run with the command<br>nohup find . -name "*.inp" -exec sh -c 'findsym {} > {}.out' \; &
AFLOW-SYM	Analysis was run from command line under Linux using binaries downloadable from https://aflowlib.org/install-aflow/<br>#!/bin/bash<br>for file in *.cif; do<br>aflow --aflowSG=1e-6 --no-scan < $file >> _output<br>done

The resulting space groups were extracted from the output text file with grep and compared to the space groups in the ICSD.
